# Epstein-Barr virus-induced up-regulation of TCAB1 is involved in the DNA damage response in nasopharyngeal carcinoma

**DOI:** 10.1038/s41598-017-03156-3

**Published:** 2017-06-12

**Authors:** Kun Wang, Yichen Ge, Chao Ni, Bomiao Cui, Jintao Du, Bo Zhang, Xiaoyu Hu, Jiao Chen, Liying Xiao, Chongkui Sun, Yan Li

**Affiliations:** 1 State Key Laboratory of Oral Diseases, West China Hospital of Stomatology, Sichuan University, Chengdu, Sichuan China; 2 Department of Prosthodontics, West China Hospital of Stomatology, Sichuan University, Chengdu, Sichuan China; 3 Department of Otorhinolaryngology–Head and Neck Surgery, West China Hospital, Chengdu, Sichuan China; 4 Department of Stomatology of University Hospital of Hubei University for Nationalities, Hubei, China

## Abstract

Telomerase Cajal body protein 1 (TCAB1), which is involved in Cajal body maintenance, telomere elongation and ribonucleoprotein biogenesis, has been linked to cancer predisposition, including nasopharyngeal carcinoma (NPC), due to its oncogenic properties. However, there are no specific reports to date on the functional relevance of TCAB1 and Epstein–Barr virus (EBV), which is considered to be a risk factor for NPC. In this study, we first examined NPC clinical tissues and found a notable overexpression of TCAB1 in EBV-positive specimens. Secondly, on a cellular level, we also observed that TCAB1 expression rose gradually along with the increased duration of EBV exposure in NPC cell lines. Additionally, EBV infection promoted cell proliferation and telomerase activity, but the activation was significantly inhibited after TCAB1 knockdown. Moreover, depletion of TCAB1 caused both cell cycle arrest and apoptosis, and suppressed the activation of ataxia telangiectasia and Rad3 related protein (ATR) induced by EBV, resulting in accumulation of DNA damage. Taken together, we here demonstrate that up-regulated expression of TCAB1, induced by EBV in the development of NPC, is involved in stimulating telomerase activity and regulating the DNA damage response within the context of EBV infection.

## Introduction

The *WRAP53* gene, located on chromosome 17p13, encodes three functional products: WRAP53α, -β, and -γ. WRAP53α is an antisense transcript that stabilises p53 by targeting the 5′-untranslated region of the p53 mRNA^1, 2^. WRAP53β, also called WDR79 or telomerase Cajal body protein 1 (TCAB1), is a scaffold protein containing WD40 repeats. Since 2009, TCAB1 has been known to be an essential component of the telomerase holoenzyme involved in telomerase assembly and Cajal body formation^3, 4^. Germline mutations in TCAB1 affecting the WD40 domain have been linked to several genetic disorders, e.g., dyskeratosis congenita, a disease associated with premature aging and cancer predisposition^5^, and spinal muscular atrophy, a neurodegenerative disorder that is a leading genetic cause of infant mortality worldwide^6^. In addition, TCAB1 dysfunction has been correlated with an elevated risk of developing a variety of sporadic tumours, including rectal, ovarian and oesophageal cancers^7–9^. Meanwhile, our previous study also implicated TCAB1 in the tumourigenesis or development of head and neck cancers^10^. Indeed, evidence to date indicated that TCAB1 possesses oncogenic properties that could facilitate tumourigenesis and tumour development. These findings imply that TCAB1 might be a potential target for early diagnosis or molecular therapy for head and neck cancers.

Nasopharyngeal carcinoma (NPC) is a malignancy associated with Epstein–Barr virus (EBV), a human γ-herpesvirus that occurs with a high incidence in East Asia, especially in Southern China^11, 12^. EBV has long been postulated to play an important role in several human malignancies including NPC^13, 14^. Previous studies have indicated that EBV up-regulates the activity of telomerase in NPC cell lines by activating several different signalling pathways, such as nuclear factor kappa B (NF-κB), c-jun N-terminal kinase (JNK), p16INK4A/pRb/E2F1, and mitogen-activated protein kinase (MAPK) pathways^15–17^. In addition, evidence also suggested that EBV would induce host genomic instability via accumulation of DNA damage^14, 18^. In the past few years, although it has been reported that EBV, as well as other oncogenic viruses, attenuates the DNA damage response (DDR) indirectly and possibly directly, the detailed regulatory mechanism remains unclear^19, 20^. DNA damage is a very frequent occurrence, and accordingly repair of such damage is critical for maintaining genome integrity and preventing tumourigenesis^21^. A recent study demonstrated that EBV infection leads to replication stress-associated DNA damage and activation of ataxia telangiectasia and Rad3 related protein (ATR) in human B cells^22^. Furthermore, up-regulation of signal transducer and activator of transcription 3 (STAT3) resulting from EBV infection was found to promote viral oncogene-driven cell proliferation and potentially result in tumourigenesis^22^. Nevertheless, the underlying oncogenic mechanisms of EBV in NPC remain enigmatic, and more specific studies are required.

In addition to the involvement in telomerase holoenzyme trafficking and assembly, TCAB1 was recently shown to be a scaffold for DNA double-strand break (DSB) repair^23^. As a novel essential regulator of the DNA DSB response, TCAB1 or WRAP53β facilitates the accumulation of the E3 ligase RNF8 to DSB sites and promotes efficient assembly of the damage repair complex; this highlights the function of TCAB1 in DDR^23^. In addition, another study demonstrated that loss of TCAB1 in epithelial ovarian cancers significantly attenuates DDR, resulting in DNA DSB accumulation and poor patient survival^8^. Although it is known that EBV infection resulted in DNA damage accumulation and attenuated the following response in NPC, whether TCAB1 that is significantly up-regulated in EBV-positive NPC samples in our study is involved in this process is unclear. Therefore, it is crucial and worth to explore the association between EBV and TCAB1 and to study the roles of TCAB1 in the tumourigenesis and development of NPC.

In the current investigation, we found that up-regulation of TCAB1 induced by EBV participates in the activation of telomerase and ATR, indicating that TCAB1 might be involved in the carcinogenic mechanism mediated by EBV in two different ways in the development of NPC.

## Results

### TCAB1 is overexpressed in EBV-positive NPC clinical specimens

**Figure 1:**
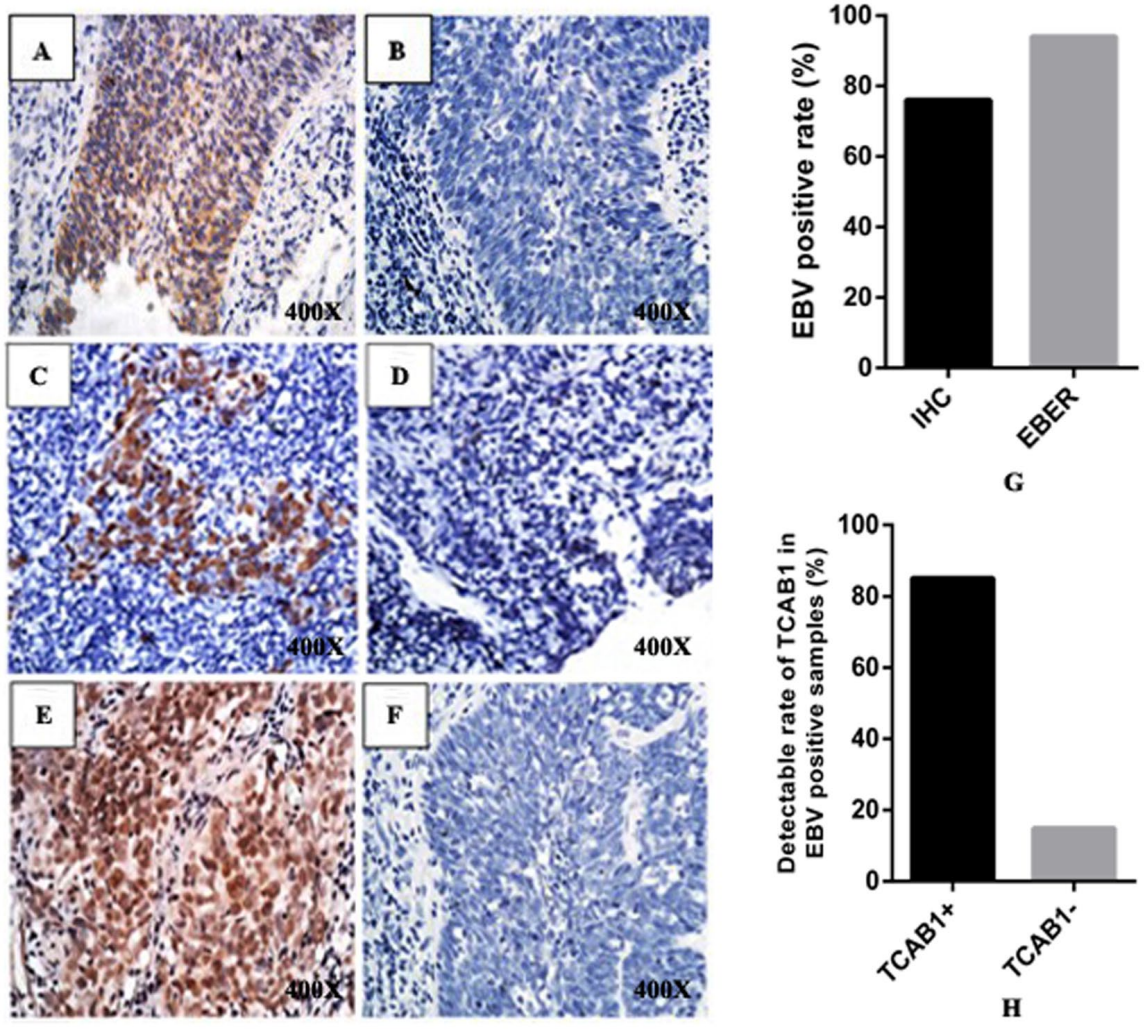
Representative photographs of LMP1 and TCAB1 expression in clinical samples of nasopharyngeal carcinoma (NPC), as detected by immunohistochemistry and *in situ* hybridisation. Typical IHC results for LMP1 ((**A**) positive (**B**) negative) in human NPC samples. Typical *in situ* hybridisation results for EBER ((**C**) positive (**D**) negative) in human NPC samples. Typical IHC results for TCAB1 ((**E**) positive (**F**) negative) in human NPC samples. (**G**) The detection rate of EBV-positive specimens by IHC and *in situ* hybridisation. (**H**) Percentage of TCAB1-positive and -negative specimens among EBV-positive samples.

To investigate EBV infection in fifty human NPC specimens, we performed immunohistochemistry (IHC) to examine expression of LMP1 and observed a positivity rate of 76% (38 of 50 samples, Fig. 1A,B). We further detected EBV-encoded RNA (EBER) by *in situ* hybridisation and found that 9 of the LMP1-negative samples were also EBV positive (Fig. 1C,D). Thus, EBV positivity was found in 47 of 50 samples. Furthermore, we assessed the expression level of TCAB1 in the NPC specimens, revealing TCAB1 overexpression in 85.1% of EBV-positive NPC biopsies (40 of 47 samples, Fig. 1E,F). Conversely, none of the EBV-negative NPC samples exhibited TCAB1 overexpression, with differences that were significant (P < 0.05, Table 1).

**Table 1:** Examination of EBV and TCAB1 in NPC clinical specimens.

Group	Number	TCAB1		P value
		Positive (%)	Negative (%)	
EBV-positive	47	40 (85.1)	7 (14.9)	<0.05
EBV-negative	3	0 (0.0)	3 (100.0)	

### Stable infection of EBV increases expression of TCAB1 in NPC cell lines

**Figure 2:**
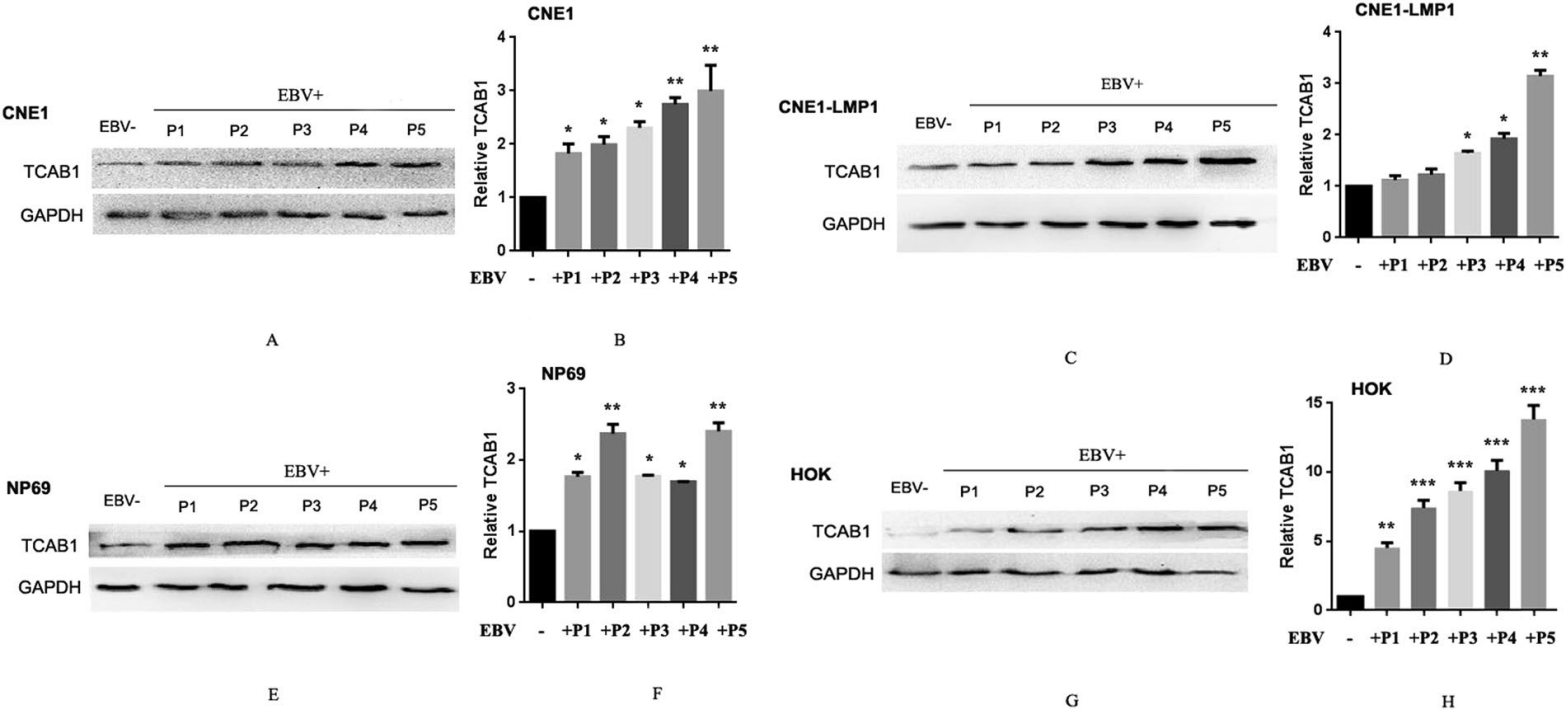
The expression level of TCAB1 is up-regulated by EBV in cell lines. (**A**,**C**,**E**,**G**) Protein level of TCAB1 in the first to fifth passages of CNE1, CNE1-LMP1, NP69 and HOK cells after EBV infection compared to EBV-negative cells. (**B**,**D**,**F**,**H**) Quantitative results of TCAB1 protein levels relative to GAPDH in each cell line. EBV+, EBV-positive; EBV−, EBV-negative. P1-5: passages 1–5 after EBV infection. The error bars represent the mean ± SD values. The statistical analysis was performed using Student’s *t* test (*P < 0.05, **P < 0.01, ***P < 0.001).

To investigate the relationship between TCAB1 up-regulation and EBV infection, the cell lines CNE1, CNE1-LMP1, NP69 and HOK were continuously exposed for three days to EBV at a multiplicity of infection (MOI) of 100. Immunofluorescence staining showed high nuclear expression of EBV-determined nuclear antigen (EBNA) 2, verifying EBV infection (Supplementary Fig. 1). Next, we collected EBV-infected cells at passages 1 to 5 and assessed the level of TCAB1 protein expression. The results showed TCAB1 to be significantly up-regulated by EBV in CNE1 and CNE1-LMP1 cells as well as in NP69 and HOK cells. Moreover, the expression level of TCAB1 increased gradually with the duration of exposure to EBV (Fig. 2A–H).

Up-regulated TCAB1 in EBV-positive CNE1 and CNE1-LMP1 cells was depleted using lentivirus carrying shTCAB1. The results of western blotting demonstrated that the level of TCAB1 protein was decreased upon lentiviral infection, whereas negative control shNC had no effect (Supplementary Fig. 2).

### Up-regulated expression of TCAB1 is involved in the promotion of telomerase activity induced by EBV

**Figure 3:**
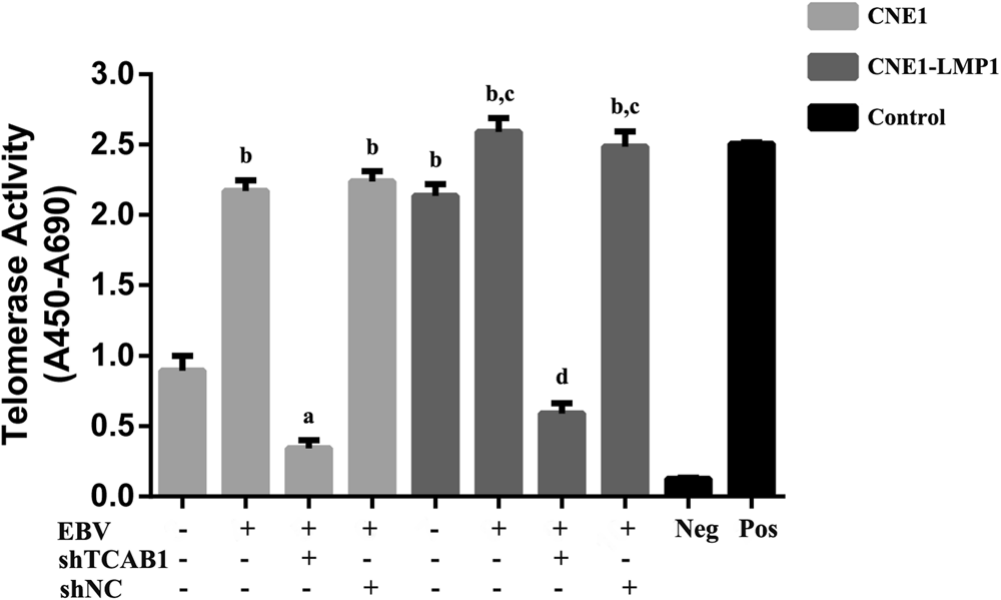
TCAB1 is involved in the promotion of telomerase activity induced by EBV. A telomerase assay was performed using telomerase PCR-ELISA. A lysate of 293 cells was heated at 85 °C for 10 min as the negative control; the unheated lysate was used as the positive control. The error bars represent the mean ± SD values. The statistical analysis was performed using Student’s *t* test (^a^P < 0.05, ^b^P < 0.01, comparing with EBV-negative CNE1; ^c^P < 0.05, ^d^P < 0.01, comparing with EBV-negative CNE1-LMP1). Telomerase was activated by EBV in CNE1 and CNE1-LMP1 cells, and inhibited by TCAB1 depletion in EBV-positive cells.

Telomerase polymerase chain reaction-enzyme-linked immunosorbent assay (PCR-ELISA) was carried out to detect telomerase activity in CNE1 and CNE1-LMP1 cells before and after EBV infection (Fig. 3). With an absorbance of 2.5 for the positive control and 0.1 for the negative control, the absorbance for CNE1 and CNE1-LMP1 cells was 1.0 and 2.2, respectively. After infection with EBV, the absorbance of CNE1 cells increased to 2.3 and that of CNE1-LMP1 to 2.7. These results indicate that EBV induces telomerase activity.

As TCAB1 is an essential component of the telomerase holoenzyme, we further investigated whether up-regulated TCAB1 expression is associated with the telomerase activity induced by EBV through examining telomerase activity in EBV-positive CNE1 and CNE1-LMP1 cells treated with shTCAB1 lentivirus. After depletion of TCAB1, the absorbance values for EBV-positive CNE1 and CNE1-LMP1 cells were 0.3 and 0.6, respectively (Fig. 3). These data implicate the involvement of up-regulated expression of TCAB1 in the activation of telomerase induced by EBV.

### Depletion of TCAB1 inhibits cell proliferation driven by EBV and induces cell cycle alteration

**Figure 4:**
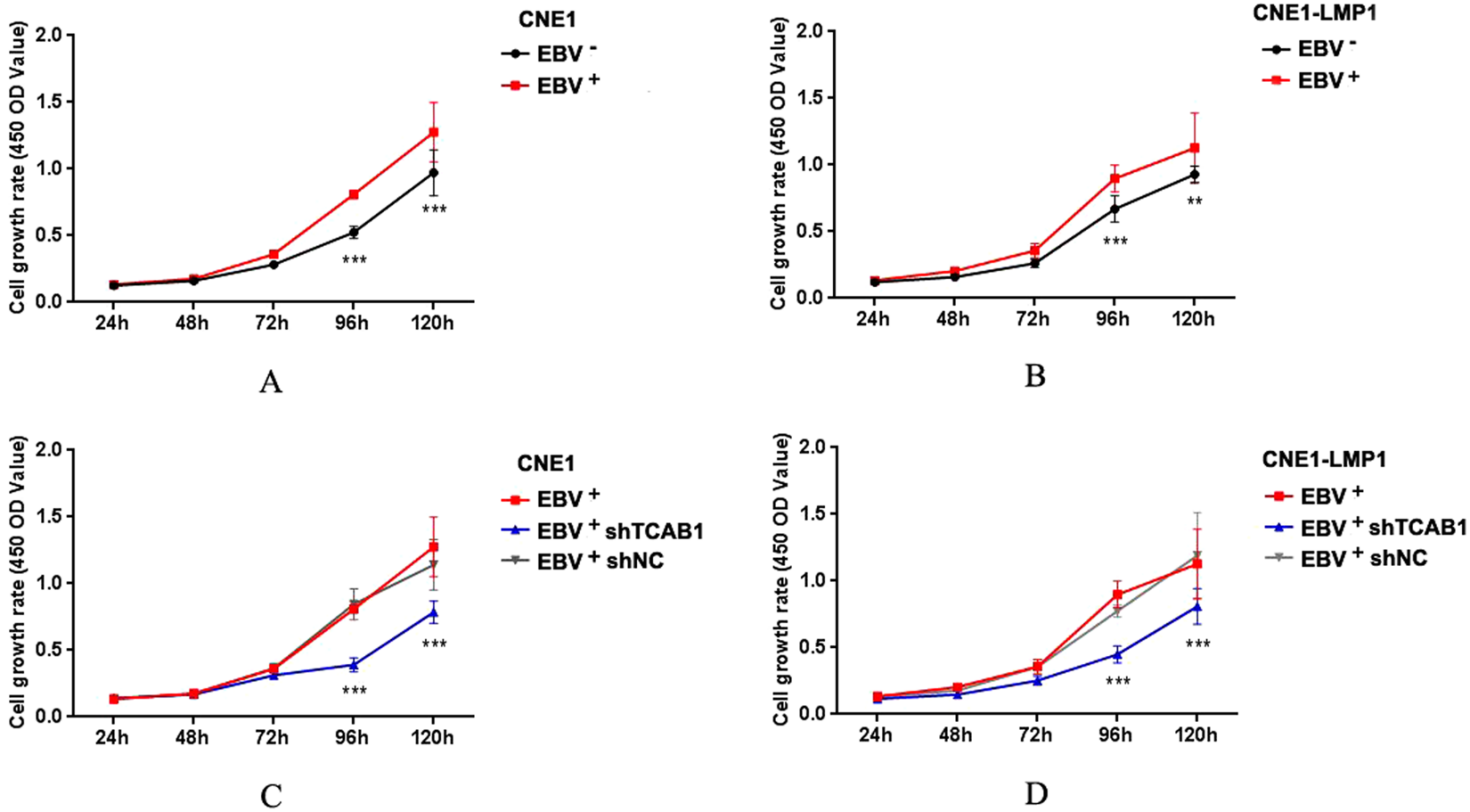
Depletion of TCAB1 reduces the proliferation of EBV-positive cells *in vitro*. (**A**) EBV promoted proliferation in CNE1 cells. (**B**) EBV promoted proliferation in CNE1-LMP1 cells. (**C**) Depletion of TCAB1 by exogenous shRNA lentivirus decreased proliferation in CNE1 cells infected with EBV. (**D**) Depletion of TCAB1 reduced proliferation in CNE1-LMP1 cells infected with EBV. The statistical analysis was performed using Student’s *t* test (**P < 0.01, ***P < 0.001).

CCK-8 assay data showed that cells infected with EBV have higher proliferation potential than EBV-negative cells, indicating that EBV promotes proliferation in NPC cell lines (Fig. 4A,B). To investigate the potential function of TCAB1 in the pathogenic mechanism of EBV in NPC, we examined the proliferation of EBV-positive CNE1 and EBV-positive CNE1-LMP1 cells treated with shTCAB1 lentivirus. Overall, cell proliferation was significantly inhibited (Fig. 4C,D). Our analyses also revealed that TCAB1 plays an essential role in the cell proliferation promoted by EBV *in vitro*, with reduction of TCAB1 expression using exogenous shRNA leading to a corresponding decrease in proliferation potential in EBV-infected cells.

**Figure 5:**
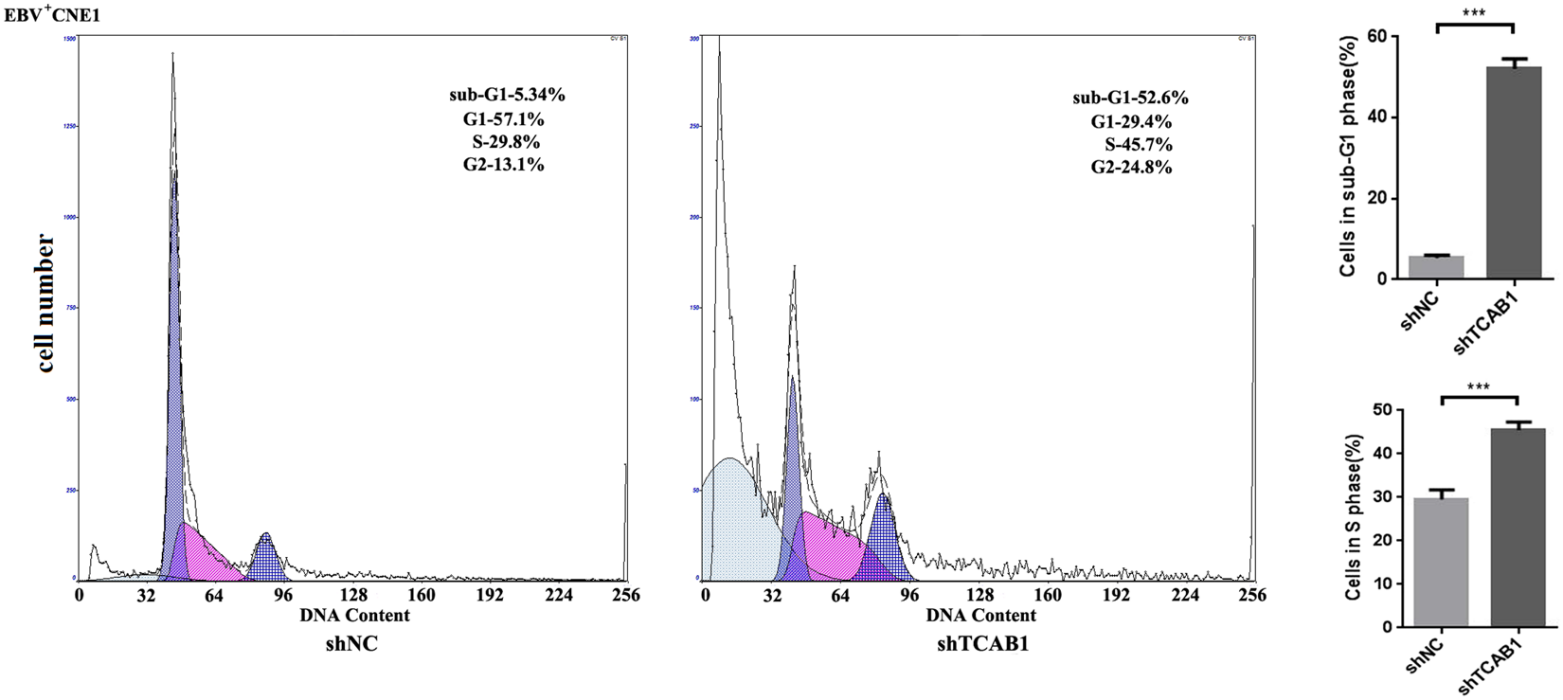
Depletion of TCAB1 promotes apoptosis and cell cycle arrest *in vitro*. Depletion of TCAB1 using exogenous shTCAB1 lentivirus inhibited cell cycle progression of EBV-positive CNE1 cells, and significantly more shTCAB1-treated cells were in sub-G1 phase than in S phase. The statistical analysis was performed using Student’s *t* test (***P < 0.001).

Flow cytometry (Fig. 5) was further performed to verify the results of the CCK-8 assay. Significantly higher cell numbers of EBV-positive CNE1 cells with TCAB1 depletion were in sub-G1 phase and S phase (52.6% and 45.7%, respectively), compared to cells treated with shNC lentivirus (5.34% and 29.8%, respectively). These findings indicate that depletion of TCAB1 induces apoptosis and cell cycle arrest in EBV-infected NPC cell lines.

### Depletion of TCAB1 results in ATR suppression and DNA damage accumulation

**Figure 6:**
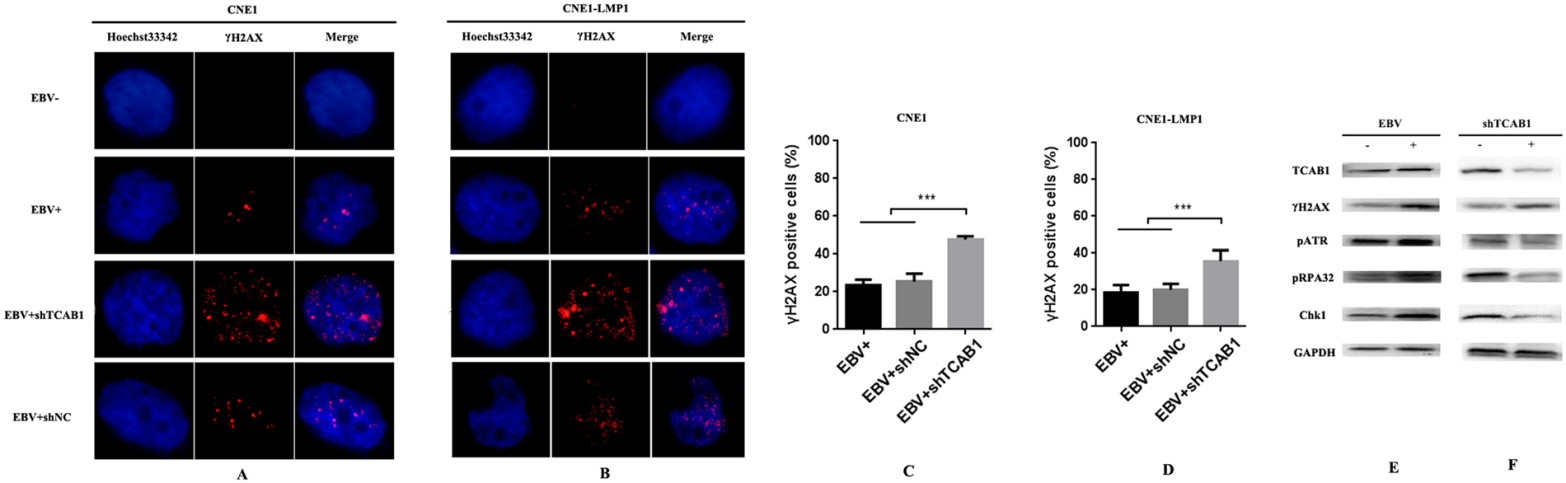
Depletion of TCAB1 inhibits phosphorylation of ATR and its respective downstream target Chk1, resulting in DNA damage accumulation following EBV infection. (**A**,**B**) CNE1 and CNE1-LMP1 cells were treated with EBV, and some cells were further infected by shTCAB1 and shNC lentivirus for 24 h, followed by immunostaining for γH2AX. (**C**,**D**) Quantification of the results in (**A**,**B**) showing the percentage of nuclei containing >10 γH2AX foci among the 200 cells counted for each experiment. The statistical analysis was performed using Student’s *t* test (***P < 0.001). (**E**) CNE1 infected with EBV was subjected to western blotting using different antibodies. (**F**) After exposure to EBV, depletion of TCAB1 interrupted ATR-to-Chk1 signalling in CNE1 cells.

We observed reduced proliferation potential and cell cycle arrest following TCAB1 knockdown. To investigate the underlying mechanism, we studied the behaviour of TCAB1 in CNE1 and CNE1-LMP1 cells after DNA damage induced by EBV. Phosphorylation of serine 139 in the C-terminal tail of the histone variant H2AX (termed γH2AX) was evaluated by immunofluorescence staining. CNE1 and CNE1-LMP1 cells were infected with EBV and then treated (or not) with shTCAB1 and shNC lentivirus, and EBV-induced foci formed by γH2AX were observed after 24 h. Importantly, shRNA-mediated knockdown of TCAB1 enhanced the number of γH2AX foci, indicating accumulation of DNA damage caused by EBV infection (Fig. 6A–D), and these results were confirmed by western blotting (Fig. 6E,F). To better characterise the role of EBV-mediated activation and increase in TCAB1 in DDR, we examined the expression of ATR, the function of which is to regulate the repair of damaged DNA replication forks. As shown in Fig. 6E, the levels of phospho (p)ATR increased after EBV infection. As a reliable marker of ATR activation^24, 25^, phosphorylation at S33 of the 32-kDa subunit of replication protein A (RPA32) was also detected following EBV infection. In contrast, after selection with puromycin, only small amounts of pATR and pRPA32 were detected in CNE1 cells treated with shTCAB1 lentivirus. As expected, the level of the critical checkpoint kinase Chk1, the downstream target of ATR that is critical for checkpoint activation upon DNA damage, was also reduced after TCAB1 depletion. These results indicate that TCAB1 is involved in ATR activation induced by EBV in response to replication stress-associated DNA damage.

### No insertion mutation in the *TCAB1* gene is detected after EBV infection

To explore the regulatory effect of EBV on TCAB1, we examined the *TCAB1* gene sequence in CNE1 and CNE1-LMP1 cells before and after EBV infection using PCR and agarose gel electrophoresis. However, no difference in the amplified *TCAB1* products was found between the EBV-negative and EBV-positive cells.

## Discussion

EBV is known as an important risk factor to push the occurrence and development of NPC through mediating a variety of cellular processes. In our current investigation, significant overexpression of TCAB1 was found in EBV-positive clinical tissues. In spite of the limited number of EBV-negative clinical samples, this finding presumedly alluded to an oncogenic property of TCAB1 and also gave a hint that up-regulation of TCAB1 might be important to the process of EBV infection during NPC tumourigenesis, prompting us to further investigate the potential association between TCAB1 and EBV at a cellular level. However, future research needs expanded sample size and randomized observation. Subsequently, we examined the expression level of TCAB1 in two NPC cells and two control cell lines before and after EBV infection, and elevated TCAB1 expression was found as the duration of EBV infection increased. This was consistent with the results for clinical specimens, suggesting that TCAB1 is up-regulated by EBV in most, if not all, NPC tissues. In addition, up-regulated expression of TCAB1 was also detected in NP69 and HOK cells after EBV infection, indicating that TCAB1 might also play a key role in the cell immortalisation triggered by EBV. Moreover, the fold increase in TCAB1 expression was higher in HOK cells than that in the other three cell lines derived from human nasopharyngeal epithelium, suggesting a dependence on cell origin, yet further investigation is required to detect the regulatory effect of EBV on TCAB1 expression in multiple cell lineages.

After infection with EBV, the proliferation potential of CNE1 and CNE1-LMP1 cells was significantly enhanced, which is consistent with a previous study^24^. In addition, our recent study showed that TCAB1 could promote CNE1 cell proliferation^10^. Based on these results, it appears that EBV and TCAB1 have a similar effect on stimulating NPC cell proliferation, which might be the result of synergy or linear superposition. To assess whether the effect of EBV and TCAB1 on NPC cells is synergic or linear superposition, TCAB1 was knocked down using shRNA lentivirus in CNE1 and CNE1-LMP1 cells. Although proliferation was significantly reduced upon TCAB1 depletion, this reduction could not be recovered in CNE1-LMP1 cells, in which LMP1, the principal oncoprotein of EBV, is stably expressed. These results indicate that EBV promotes proliferation in NPC cells by enhancing the expression of TCAB1.

Several studies have demonstrated that EBV infection and EBV gene expression results in genomic instability, facilitating the development of NPC^25, 26^. As an oncogenic virus, EBV can modulate components of the DDR pathway, particularly the histone variant H2AX, to promote oncogenesis. Phosphorylated H2AX, a mediator of DDR, was found to be associated with EBV infection in NPC cells^27^. In the present study, NPC cells exhibited γH2AX expression upon EBV infection, consistent with the results of a previous study^28^. Moreover, our findings demonstrate that EBV infection leads to activation of ATR; a similar effect of EBV was also detected in B lymphocytes^22^. In addition, TCAB1 was recently reported to be an essential regulator of DDR in an ataxia telangiectasia-mutated (ATM)-dependent manner, promoting the interactions necessary for an appropriate biological response^23^. Nonetheless, few studies have reported the roles of TCAB1 in the ATR/Chk1 pathway. Here, we found that knockdown of TCAB1 blocked the activation of ATR induced by EBV and interrupted ATR-to-Chk1 signalling. Thus, accumulation of cells in S phase may be attributed to aggravation of the DNA damage caused by suppression of TCAB1. Our findings support the results of a recent study, which found that TCAB1 interacts with γH2AX at sites of DNA damage in an ATR-dependent manner^29^. In the future, it would be very interesting to investigate the exact role of TCAB1 in DDR in EBV-associated tumours.

Induction of EBV elicits DDR with a delay or arrest of cell cycle progression, and activation of ATM signal transduction has been well studied^20, 30^. In contrast, there are few reports to date regarding activation of the ATR replication checkpoint pathway. One recent study found that STAT3 impairs Chk1 function to relax the intra-S phase checkpoint during EBV-driven cell proliferation^22^. In our study, accumulation of cells in S phase was also detected after suppression of TCAB1 in EBV-infected cells. Together with the observation of inhibited phosphorylation of ATR and its respective downstream target Chk1 induced by TCAB1 depletion, our findings suggest that TCAB1 is involved in intra-S phase DDR via the ATR-Chk1 pathway.

Furthermore, we suggest that aggravation of DNA damage might be a direct reason for the observed rapid and extensive apoptosis that occurs after TCAB1 silencing. As we know, TCAB1 has been identified as an essential factor for Cajal body maintenance, which is not imperative for telomerase biogenesis and function in human cancer cells^31^. Even though many processes in this organelle are important for survival, Cajal bodies *per se* are not essential for viability of cells. Also, previous studies indicated that Cajal bodies did not trigger apoptosis upon disruption^32–34^, suggesting that other functions of TCAB1 are essential for cancer cell survival. In general, telomerase dysfunction results in critical shortening of telomeres during progressive cell divisions. This time period is called the lag phase about decades cell divisons^35^. Eventually, the DDR triggered by critically shortened telomeres elicits an irreversible cell cycle arrest termed replicative cellular senescence^36^. Our results revealed rapid and massive apoptosis in EBV-infected cells after transfection of shTCAB1 lentivirus. This might depend on the accumulation of DNA damage rather than on the regulation of telomere shortening following a decrease to a certain threshold. The result may be the direct induction of telomerase dysfunction or disruption of the shelterin complex, eventually causing cell cycle arrest and apoptosis^37^.

In NPC cells, EBV may induce oncogenic development by interfering with several intracellular mechanisms^38–40^. One such mechanism by which EBV induces cell immortalisation and malignant transformation is via activation of telomerase during cancer development^41, 42^. A number of studies have focused on the fact that EBV regulates telomerase activity by promoting expression and phosphorylation of human telomerase reverse transcriptase (hTERT), the key determinant of the telomerase activity^12, 43^. Recently, TCAB1 was identified as a subunit of the telomerase holoenzyme, and it was found to be important for telomere maintenance in human cancer cells^3^. It is possible that factors involved in enhanced telomerase expression, such as EBV, also contribute to TCAB1 overexpression in the development of NPC. Our data presented here show that in NPC cells, TCAB1 might play an important role in the telomerase activation induced by EBV. However, we did not find an insertion in the *TCAB1* gene sequence after EBV infection in NPC cell lines. Regardless, we cannot yet rule out the possible influence of EBV on TCAB1 at the genetic level, which remains to be further investigated.

In addition, TCAB1 also has a role to guide a class of RNA molecules referred to as small Cajal body-specific RNAs (scaRNAs) to Cajal bodies, which are required for catalyzing post-transcriptional modifications of the snRNA component of small nuclear ribonucleoproteins (snRNPs)^4^. Nevertheless, the exact manner in which the different functions and regulators of TCAB1 are coordinated is not yet clear. TCAB1 has now been shown to associate specifically with the CAB box of scaRNAs and promote their targeting to Cajal bodies^4, 5^. However, the factors responsible for this targeting long remained unknown. It is worth studying for the molecular mechanism of splicing regulation of the overexpressed TCAB1 induced by EBV. Therefore, in the future research, we will further probe into the changes in the splicing capacity under the guidance of TCAB1 in the pathogenesis of NPC.

In summary, our findings give new insight into the role of TCAB1 in human NPC, providing the first evidence that EBV enhances expression of TCAB1 *in vitro*. Furthermore, we identify TCAB1 as a novel player in DDR following EBV infection, indicating the regulation of TCAB1 in the DNA damage resulting from EBV oncogene-induced replication stress. This might be a predominant cause of the overexpression of TCAB1 in NPC and a novel mechanism by which EBV drives malignancy.

## Methods

### Collection of NPC clinical specimens

A total of fifty paraformaldehyde-fixed paraffin-embedded NPC tissues with histopathology reports were collected. All clinical specimens were achieved from Sichuan Province Tumour Hospital during 2011 to 2012. This study was approved by the local ethics committee of Sichuan University. Informed consent was obtained from all study participants. The experiments were carried out in accordance with approved guidelines.

### Immunohistochemistry and *in situ* hybridisation

Immunohistochemical analyses of pathological specimens were performed as described previously^10^. Slides were incubated with a primary antibody against TCAB1 (1:200 dilution, Novus, USA) or LMP1 (1:100 dilution, Santa Cruz Biotechnology, USA) and detected using the ChemMateTM EnVision kit (Dako, Carpinteria, CA). Immune reactivity was analysed and quantified using imageScope software (Aperio, Technology, Vista, CA). For LMP1-negative samples, EBER was detected to further examine EBV infection with the *in situ* hybridisation technique according to the manufacturer’s recommended protocol (Pan Path, Amsterdam).

### Cell lines, cell culture and EBV isolation

The following established cell lines were used in this study: two NPC cell lines, CNE1 (an EBV-negative, low-differentiated NPC cell line) and CNE1-LMP1 (a stable LMP1-integrated NPC cell line); NP69 (an immortalised normal human nasopharyngeal epithelial cell line); HOK (a human oral keratinocyte) and B95-8 (a marmoset EBV-immortalised B-cell line). CNE1 and B95-8 cells were obtained from the State Key Laboratory of Oral Diseases. CNE1-LMP1 and NP69 cells were purchased from the Cancer Research Institute at Xiangya School of Medicine, Central South University, China. HOK cells were provided by Dr. Qianming Chen and were obtained from the American Type Culture Collection (ATCC, 11303). CNE1, CNE1-LMP1 and B95-8 cells were cultured in RPMI-1640 (HyClone, Logan, UT, USA) with 10% foetal bovine serum (Gibco, Grand Island, NY, USA) and 1% penicillin-streptomycin (Thermo Fisher Scientific, Waltham, MA USA). NP69 cells and HOK cells were maintained in serum-free keratinocyte medium supplemented with human recombinant epidermal growth factor (0.1–0.2 ng/mL) and bovine pituitary extract (20–30 μg/mL) (Gibco). All cells were cultured in a humidified incubator at 37 °C with 5% CO_2_.

To isolate EBV from B95-8 cells, multigelation and ultrasonication were used with cells in late log phase. The virus solution was obtained after filtration of the treated culture fluid using a 0.22-μm-pore-size filter. The virus titre was calculated to be 1.0 × 10^9^ copies/mL using real-time PCR and used for experiments.

### Immunofluorescence

Cells were grown on sterilised coverslips and fixed with 4% paraformaldehyde (PFA) for 15 min at room temperature. The cells were then permeabilised with 0.2% Triton X-100 for 5 min followed by 30 min of blocking in 4% foetal bovine serum. The cells were incubated with primary antibodies against EBNA2 (1:200 dilution, Abcam, USA) or γH2AX (1:200 dilution, Signalway Antibody, USA) for 2 h and then with an Alexa Fluor 594 dye-conjugated secondary antibody (1:500 dilution, Thermo Fisher) for 1 h before counterstaining the nucleus with Hoechst 33342 (Sigma-Aldrich, USA) at room temperature. Finally, the slides were covered with fluorescence mounting medium (Dako) and photographed under a fluorescence microscope (Olympus, Tokyo, Japan).

### Western blotting

Cells were trypsinised, harvested, washed, and lysed in ice-cold Radio Immunoprecipitation Assay buffer (RIPA lysis buffer, Beyotime, Jiangsu, China). Western blotting was performed according to standard procedures. Primary antibodies against TCAB1 (1:4000 dilution, Novus), γH2AX (1:1000 dilution, Santa Cruz), pATR (1:5000 dilution, Abcam), pRPA32 (1:1000 dilution, Santa Cruz) and Chk1 (1:1000 dilution, Santa Cruz) were diluted in 5% BSA. GAPDH (1:1000 dilution, Santa Cruz) was used as an internal control.

### Lentivirus assembly and infection

The shTCAB1 lentivirus was assembled using pCMV-dR8.2 dvpr (Addgene), pCMV-V-SVG (Addgene) and shTCAB1 (shRNA, targeting CACCCAACCTGAGAACTTCTT, Sigma Aldrich). The shNC control vector was constructed using a scramble shRNA sequence. After infection with EBV, CNE1 and CNE1-LMP1 cells were infected by shTCAB1 lentivirus using polybrene (5 μg/mL, EMD Millipore Temecula, CA, USA) and selected using puromycin (Thermo). shNC lentivirus was used as a negative control.

### Telomerase activity assay

Telomerase activity was measured using the telomerase PCR-ELISA assay (Roche Molecular Biochemicals, Mannheim, Germany) according to the manufacturer’s instructions. Cells were washed in phosphate-buffered saline (PBS), trypsinised, counted, and centrifuged at 3000× g for 10 min. The pelleted cells were resuspended in lysis reagent, incubated on ice for 30 min at 4 °C, and centrifuged at 16000× g for 20 min at 4 °C. Cell extracts from 10^3^ cells were then used in the telomerase PCR-ELISA assay. Positive controls for telomerase activity consisted of extracts of 293 cells. Negative controls were cell extracts heat-treated for 10 min at 85 °C to degrade the RNA component of telomerase. Telomeric repeat amplification was performed as follows: 30 min at 25 °C for primer elongation; 5 min at 94 °C for telomerase inactivation; 30 cycles of 30 s at 94 °C, 30 s at 50 °C, and 90 s at 72 °C for amplification; a final cycle for 10 min at 72 °C. Aliquots of the PCR products were denatured, hybridised to a digoxigenin (DIG)-labelled probe complementary to telomere repeat sequences, and incubated with an anti-DIG-peroxidase antibody in a streptavidin-coated microtitre plate. The PCR products were then visualised by colour development after adding a peroxidase substrate. Absorbance was measured at 450 nm with a reference wavelength at 690 nm using a Thermo Scientific Varioskan Flash (Thermo Scientific). The results are expressed as the average of duplicate samples normalised to their respective heat-treated controls.

### CCK-8 assay for cell proliferation

Cell proliferation was measured using Cell Counting Kit-8 (CCK-8, Dojindo, Japan). Briefly, cells were seeded in 96-well plates at a density of 10^3^ per well, and duplicates of 4 wells each were prepared for each time point. Cell viability was assessed every 24 h after seeding. Absorbance at 450 nm was measured using a Thermo Scientific Varioskan Flash (Thermo Scientific).

### Flow cytometry to examine the cell cycle

Dissociated cells were fixed and permeabilised overnight with 70% pre-cooled ethanol at 4 °C; the cells were then incubated with RNase A at 37 °C for 30 min and stained with propidium iodide (PI) at 4 °C for 30 min (Cell Cycle Detection Kit, KeyGEN, Jiangsu, China). A cell cycle assay was performed using a flow cytometer (Beckman Coulter, USA).

### Primer design and detection of TCAB1 insertion

Genomic DNA was extracted from CNE1 and CNE1-LMP1 cells before and after infection with EBV using the Blood and cell culture DNA mini kit (Qiagen, Valencia, CA, USA). The primers used for detecting the *TCAB1* gene are provided in Supplementary Table 1. The amplification conditions were denaturation at 94 °C for 30 s (3 min for the first cycle), annealing at 51 °C for 30 s and extension at 72 °C for 1 min (10 min for the last cycle) for 30 cycles. The PCR product was separated by agarose gel electrophoresis.

### Statistical analysis

SPSS 19.0 software (Chicago, IL, USA) and GraphPad Prism software, version 6 (San Diego, CA, USA) was used for statistical analysis. Student’s *t*-test was used for comparisons of two independent groups. P value less than 0.05 was considered statistically significant.

## Electronic supplementary material

Supplementary information
