# Supplementary material for: Epstein-Barr virus-induced up-regulation of TCAB1 is involved in the DNA damage response in nasopharyngeal carcinoma
Source: Sci Rep. 2017 Jun 12;7:3218. doi: 10.1038/s41598-017-03156-3 (PMC5468285; doi:10.1038/s41598-017-03156-3)
Supplement: Supplementary file 1 — Supplementary information [file 41598_2017_3156_MOESM1_ESM.pdf]

Epstein-Barr virus-induced up-regulation of TCAB1 is involved in the DNA damage response in nasopharyngeal carcinoma

Kun Wang[1†], Yichen Ge[1†], Chao Ni[1, 2†], Bomiao Cui[1], Jintao Du[3], Bo Zhang[1, 4], Xiaoyu Hu[1], Jiao Chen[1], Liying Xiao[1*], Chongkui Sun[1*], Yan Li[1*]

[1]State Key Laboratory of Oral Diseases, West China Hospital of Stomatology, Sichuan University, Chengdu, Sichuan, China

[2]Department of Prosthodontics, West China Hospital of Stomatology, Sichuan University, Chengdu, Sichuan, China

[3]Department of Otorhinolaryngology–Head and Neck Surgery, West China Hospital, Chengdu, Sichuan, China

[4]Department of Stomatology of University Hospital of Hubei University for Nationalities, Hubei, China

[†]Equal contributors

[*]Corresponding author: Yan Li, e-mail: feifeiliyan@163.com, Chongkui Sun, e-mail: cksun@scu.edu.cn, or Liying Xiao, e-mail: klobme@163.com；State Key Laboratory of Oral Diseases, West China Hospital of Stomatology, Sichuan University, Chengdu 610041, China.

Supplementary materials

Supplementary Figure 1. EBNA2 was positive staining in cell lines after infection with EBV. CNE1, CNE1-LMP1, NP69 and HOK cells were detected with indirect immunofluorescence staining with an antibody specific to EBNA2 after EBV infection. The nuclei of the cells were stained with Hoechst33342. EBV+, EBV-positive; EBV-, EBV-negative.

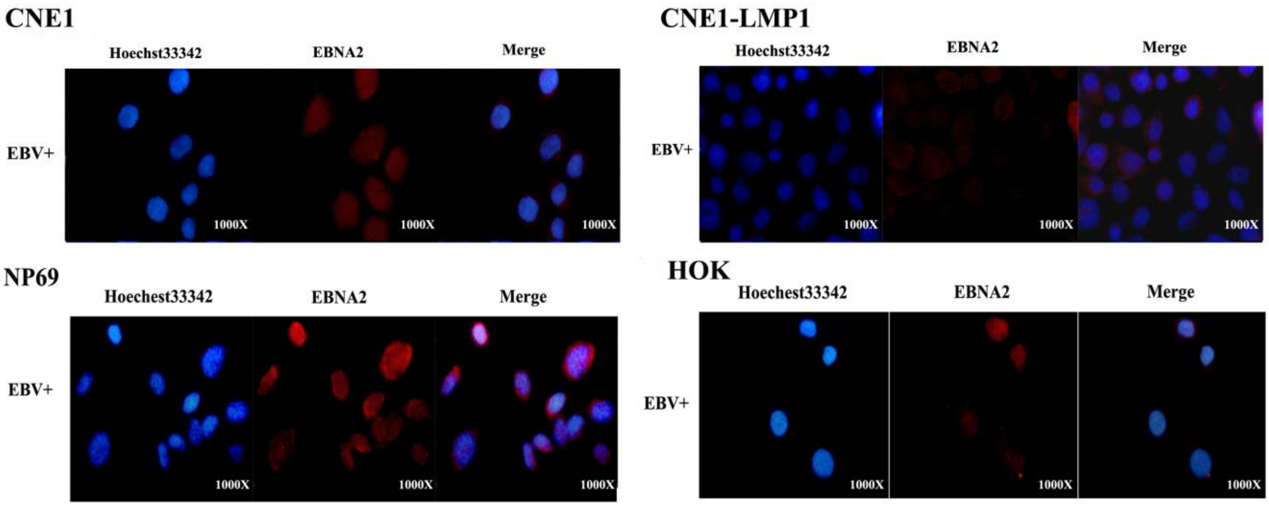

Supplementary Figure 2. Protein level of TCAB1 decreased efficiently after shTCAB1 lentivirus treatment. Using exogenous shTCAB1 lentivirus significantly depleted protein level of TCAB1 in CNE1 and CNE1-LMP1. Statistical analysis was determined by Student's *t* test (*** $P < 0.001$).

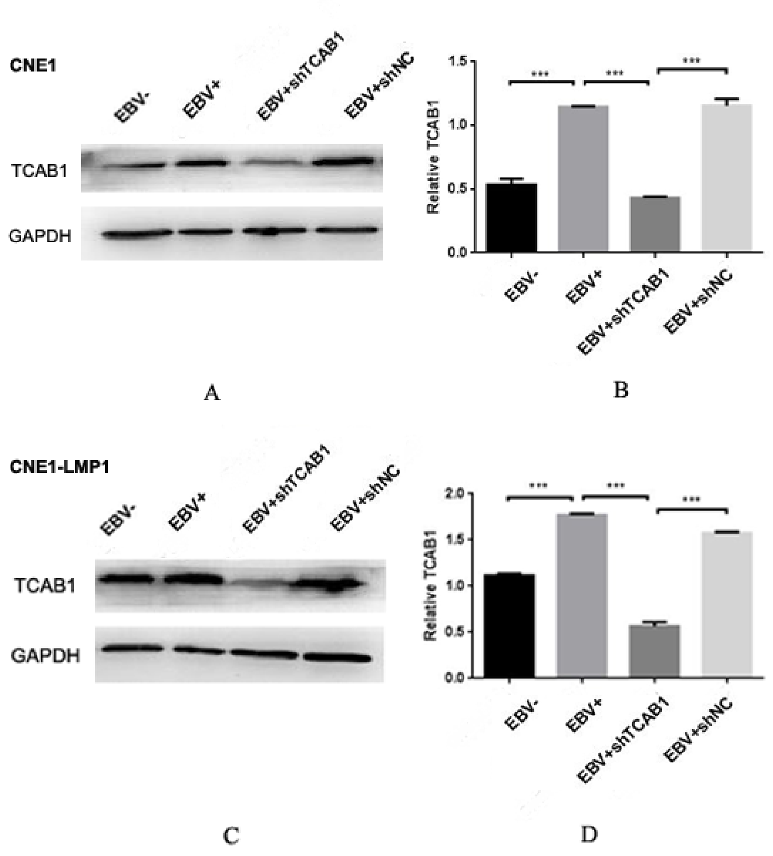

Supplementary Table 1. Primer design for the amplification of *TCAB1* gene.

Primers	Sequences	Amplified fragment length
Primer 1	F1-70: TGTAATCCCAGCACTTTG<br>R1-474: CTGGTATCTTTCCTGTATG	404bp
Primer 2	F2-360:AGCCCTATACTCCAACTG<br>R2-858: CTCTTCGAGTCTTTTACCT	451bp
Primer 3	F3-784: ATGTATAAAGCAGTCCCACA<br>R3-1234: GAGTTGTTTCCCATTTGC	499bp
Primer 4	F4-1118: TCGCAATTCCCCTCCTAA<br>R4-1579: ACGTGGTGAATCCCCGTC	462bp
Primer 5	F5-1488: CTGCCTCACCCTCCCGAATA<br>R5-2121: TCCAGACGCAGGCTGAACG	634bp
